# Improved Methods for Deamination-Based m^6^A Detection

**DOI:** 10.3389/fcell.2022.888279

**Published:** 2022-04-27

**Authors:** Huanyu Zhu, Xinhe Yin, Christopher L. Holley, Kate D. Meyer

**Affiliations:** ^1^ Department of Biochemistry, Duke University School of Medicine, Durham, NC, United States; ^2^ Department of Molecular Genetics and Microbiology, Duke University School of Medicine, Durham, NC, United States; ^3^ Department of Medicine, Duke University Medical Center, Durham, NC, United States; ^4^ Department of Neurobiology, Duke University School of Medicine, Durham, NC, United States

**Keywords:** m^6^A, epitranscriptome, DART-seq, RNA modification, RNA biology

## Abstract

*N* ^6^-methyladenosine (m^6^A) is a critical regulator of gene expression and cellular function. Much of our knowledge of m^6^A has been enabled by the identification of m^6^A sites transcriptome-wide. However, global m^6^A profiling methods require high amounts of input RNA to accurately identify methylated RNAs, making m^6^A profiling from rare cell types or scarce tissue samples infeasible. To overcome this issue, we previously developed DART-seq, which relies on the expression of a fusion protein consisting of the APOBEC1 cytidine deaminase tethered to the m^6^A-binding YTH domain. APOBEC1-YTH directs C-to-U mutations adjacent to m^6^A sites, therefore enabling single nucleotide-resolution m^6^A mapping. Here, we present an improved version of DART-seq which utilizes a variant of the YTH domain engineered to achieve enhanced m^6^A recognition. In addition, we develop *in vitro* DART-seq and show that it performs similarly to cellular DART-seq and can map m^6^A in any sample of interest using nanogram amounts of total RNA. Altogether, these improvements to the DART-seq approach will enable better m^6^A detection and will facilitate the mapping of m^6^A in samples not previously amenable to global m^6^A profiling.

## Introduction

*N* ^6^-methyladenosine (m^6^A) is the most abundant internal mRNA modification and plays important roles in multiple aspects of mRNA regulation, including translation, splicing, and stability (Meyer and Jaffrey, 2017; Zaccara et al., 2019). m^6^A is deposited at RAC sites (R = A or G) by a methyltransferase complex composed of METTL3, METTL14, WTAP and other cofactors and is enriched in proximal 3′UTRs and in the vicinity of the stop codon (Meyer et al., 2012; Meyer and Jaffrey, 2017; Roundtree et al., 2017; Shi et al., 2019; Zaccara et al., 2019; He and He, 2021). Consistent with its broad roles in gene expression control, m^6^A is important for several physiological processes, including stem cell fate decisions, learning and memory, and immune responses (Shi et al., 2018; Winkler et al., 2019; Zhang et al., 2020). Additionally, abnormal regulation of m^6^A or its regulatory proteins contributes to a variety of human diseases, including several cancers (Chen et al., 2019; He et al., 2019; Gu et al., 2020; Wang et al., 2020). Thus, being able to identify the RNAs that contain m^6^A in cells or tissues of interest is critical for enhancing our understanding of how this modification contributes to cellular function and for elucidating the impact that it has on human disease.

Traditional m^6^A profiling approaches have used m^6^A antibodies to immunoprecipitate methylated RNAs (Hafner et al., 2010; Dominissini et al., 2012; Meyer et al., 2012; Linder et al., 2015; Hsu and He, 2019). Such methods have been critical for our understanding of m^6^A distribution and regulation, but they suffer from limitations that include cross-reactivity with other modifications and the requirement for large amounts of RNA. Recently, a variety of antibody-free methods have been developed (Owens et al., 2021), but these also generally require large amounts of input material. To overcome these limitations, our group recently developed DART-seq (deamination adjacent to RNA modification targets), which utilizes a fusion protein consisting of the m^6^A-binding YTH domain tethered to the cytidine deaminase APOBEC1 (hereafter APO1) to direct C-to-U editing at m^6^A-adjacent cytidines (Meyer, 2019). DART-seq relies on a simple RNA-seq readout and can therefore identify m^6^A sites at single-nucleotide resolution using low amounts of RNA, including in single cells (Tegowski et al., 2022a). However, one limitation of DART-seq is that it relies on expression of the APO1-YTH fusion protein in cells of interest, which may not always be possible or desirable. To address this, we previously developed an *in vitro* DART-seq approach (Meyer, 2019), but this strategy used a relatively crude APO1-YTH protein preparation and exhibited reduced sensitivity compared to cellular DART-seq. Thus, further optimization of the *in vitro* DART-seq approach is needed for it to be an effective tool for global m^6^A mapping.

Here, we perform a systematic optimization of the major components of the DART fusion protein in an attempt to maximize m^6^A detection sensitivity. We find that introducing a D422N mutation into the YTH domain of the DART protein leads to improved m^6^A binding and m^6^A detection transcriptome-wide. In addition, we find that substituting APO1 with the catalytic domain of ADAR containing a hyperactive E488Q mutation (ADARcd) characterized previously (Rahman et al., 2018) enables identification of methylated RNAs based on A-to-I editing and therefore provides an alternative approach for DART-seq-based m^6^A profiling. Finally, we develop an improved version of *in vitro* DART-seq using the APO1-tethered DART protein and demonstrate its ability to identify m^6^A sites with single-nucleotide resolution transcriptome-wide from ultra-low amounts of total RNA. Altogether, the tools developed here enhance the sensitivity of the original DART-seq approach and also provide new strategies for the detection of m^6^A in virtually any sample of interest.

## Methods

### Plasmids

DART protein variants used for cellular DART-seq (A3A-YTH, A3C-YTH, huAPO1-YTH, AID-YTH, rZDD-YTH, APO1-YTH^DF1^, APO1-YTH^DF1(D401N)^, and APO1-YTH^D422N^), were cloned into the pCMV-APOBEC1-YTH plasmid (Addgene #131636) in place of APOBEC1 or the YTH domain as indicated using Gibson Assembly (NEB). ADARcd-YTH^D422N^ and ADARcd-YTH^mut^ plasmids were generated by replacing APOBEC1 from the pCMV-APOBEC1-YTH and pCMV-APOBEC1-YTH^mut^ plasmids (Addgene #131636 and #131637) with ADARcd containing a hyperactivating E488Q mutation (Addgene #139686) using Gibson Assembly (NEB). *In vitro* DART-seq proteins (APO1-YTH, APO1-YTH^mut^, APO1-YTH^D422N^, and APOBEC1 alone) were cloned into the PET-His6-MBP-TEV LIC plasmid (Addgene #29656) by ligation independent cloning using a T4 DNA Polymerase (NEB). YTH domain of human YTHDF2 was cloned into the PET-His6-MBP-TEV LIC cloning vector (Addgene #29656) with Gibson Assembly (NEB).

### Cell Culture

HEK293T cells were obtained from the American Type Culture Collection (ATCC) and cultured in Dulbecco’s Modified Eagle’s Medium (DMEM) with 4.5 g/L glucose, L-glutamine, and sodium pyruvate (Corning) supplemented with 10% (v/v) fetal bovine serum (Avantor Seradigm) and Penicillin-Streptomycin (Gibco). Cells were cultured in a humidified incubator maintained at 37°C with 5% CO_2_.

### Cellular DART-Seq

Three independent plating and RNA isolation experiments were performed using HEK293T cells transiently expressing APO1-YTH, APO1-YTH^mut^, A3A-YTH, A3C-YTH, huAPO1-YTH, AID-YTH, rZDD-YTH, APO1-YTH^DF1^, APO1-YTH^DF1(D401N)^, APO1-YTH^D422N^, ADARcd-YTH^D422N^, ADARcd-YTH^mut^, and ADARcd. DART constructs were transiently transfected into HEK293T cells using Lipofectamine 2000 according to the manufacturer’s protocol (Thermo Fisher). DART protein were expressed in HEK293T cells for 24 h. Cells were then briefly rinsed with cold 1X PBS and removed from the culture plate using a cell scraper. Total RNA was isolated using Trizol (Invitrogen) according to the manufacturer’s instructions and subsequently treated with DNase I (NEB) for 15 min at 37°C to remove possible DNA contamination. RNA was then purified using ethanol precipitation and used for downstream analysis with either Sanger sequencing or next-generation sequencing.

### Treatment of HEK293T Cells With STM2457

HEK293T cells were cultured to 40% confluency in Dulbecco’s Modified Eagle’s Medium (DMEM) as described above. 10 μM of STM2457 (WuXi AppTec) dissolved in water was then added to the culture media. Cells were incubated with this treatment for 72 h. Cellular DART-seq with ADARcd-YTH^D422N^ were conducted for STM2457 treated HEK293T cells through transient transfection 48 h after the start of 10 µM of STM2457. ADARcd-YTH^D422N^ construct was expressed in treated cells for 24 h, and the cells were cultured in media containing 10 µM of STM2457 for a total 72 h treatment.

### *In vitro* DART-Seq

Purified APO1-YTH^D422N^, APO1-YTH and APO1-YTH^mut^ proteins were purified as previously described (Tegowski et al., 2022b). DART proteins were expressed in One Shot™ BL21 (DE3) pLysE Chemically Competent *E. coli* (Invitrogen) through auto-induction. Bacterial lysate were then collected and processed using the Qproteome Bacterial Protein Prep Kit (Qiagen) following manufacturer protocol. DART protein was then affinity purified from lysate with Ni-NTA agarose beads (Gold Biotechnology) packed in a Poly-prep chromatography column (Biorad). The *In vitro* DART-seq assays were performed by incubating 250 ng of purified DART protein with 50 ng of total HEK293T cell RNA in DART buffer (10 mM Tris-HCl (pH 7.4), 50 mM KCl, 0.1 µM ZnCl_2_) and 1 µl RNaseOUT (Invitrogen) in a total volume of 50 µl for 4 h at 37°C. For *in vitro* DART-seq assays using the YTH blocking negative control, RNA was pre-incubated with 1 µg of purified YTH domain and 1 µl RNaseOUT in 30 µl volume in water at 37°C for 1 h with rotation. YTH blocked RNA samples were then incubated with 250 ng of purified APO1-YTH^D422N^ protein with 50 ng of total HEK293T cell RNA in DART buffer (10 mM Tris-HCl (pH 7.4), 50 mM KCl, 0.1 µM ZnCl_2_) and 1 µl RNaseOUT (Invitrogen) in a total volume of 50 µl for 4 h at 37°C. RNA was isolated with the Qiagen RNeasy Plus Mini Kit (Qiagen) and stored at −80°C before thawed for downstream analysis with either Sanger sequencing or next generation sequencing.

### Western Blotting

Cells were quickly rinsed with cold 1x PBS and scraped from culture plates. Cells were then pelleted by centrifugation at 1,000 × g for 3 min at 4°C. Cell pellets were resuspended in lysis buffer [25 mM Tris-HCl, pH7.4; NaCl 150 mM; Triton X-100 1% (v/v); sodium dodecyl sulfate 0.1% (v/v); complete proteinase inhibitor cocktail (Sigma-Aldrich)] and incubated on ice for 10 min. Lysates were then centrifuged at 13,000 × g for 15 min at 4°C. The supernatant was transferred to a new tube. Samples for SDS-PAGE were then prepared at a final concentration of 1 μg/μl total protein in 1 × NuPAGE LDS Sample Buffer (Invitrogen) and 0.1 M DTT (VWR). Samples were run on 4–12% SDS-PAGE gels (Invitrogen) and transferred for 60 min at 100 V in Towbin transfer buffer [25 mM Tris Base, 192 mM Glycine, 20% methanol (v/v)] to a PVDF membrane (GE Amersham). After transferring, the membrane was blocked in PBST [PBS with 0.1% Tween 20 (Sigma-Aldrich)] with 5% milk (w/v) (Quality Biological) for 1 h at room temperature. Primary antibodies, anti-βactin (Genscript), or anti-HA (Cell Signaling Technology) were incubated with the blots overnight at 4°C. The membrane was washed 3 times with PBST before the secondary antibody was added for 1 h at room temperature in PBST. Anti-rabbit-HRP secondary (Fisher Scientific) was used at 1:10,000 dilution, while anti-mouse-HRP secondary (Fisher Scientific) was used at 1:2,500. The membrane was then washed 3 times with PBST for 5 min. The western blot was visualized using Amersham ECL Prime Reagent (Amersham) and imaged on a Chemidoc MP (BioRad).

### RNA Pulldown Assays

An appropriate volume of magnetic Streptavidin beads (Fisher), 20 μl per pulldown reaction was aliquoted, equilibrated with 480 μl of Binding Buffer [10 mM Tris, pH 7.5, 1.5 mM MgCl2, 150 mM KCl, 0.5 mM DTT, 0.05% (v/v) NP-40 substitute]. Magnetic Streptavidin beads was then further divided based on usage for each of the purified DART protein variants described above (Control: no RNA oligo, A: 5′-biotin unmodified RNA oligo, m^6^A: 5′-biotin m^6^A-modified RNA oligo). Magnetic Streptavidin beads were then batch-incubated with 2 μg of each RNA oligo in Binding Buffer + 100 U/ml RNase inhibitor for 1 h at 4°C on a rotator.

Concurrently, 500 ng of purified DART proteins variants (APO1-YTH, APO1-YTH^DF1^, APO1-YTH^DF1(D401N)^, and APO1-YTH^D422N^) were resuspended in an 250 µl of Binding Buffer and kept on ice. 20 μl of the resuspension were taken as input for each sample for downstream Western blotting analysis.

After incubation, Streptavidin beads were washed twice with 360 μl of Binding Buffer (clearing with magnetic stand each time) to remove any unbound RNA oligo from solution (control/mock samples were treated and washed identically), and aliquoted and resuspeded in 20 μl/sample of Binding Buffer. Finally 20 μl of RNA bait attached Streptavidin beads were incubated with resuspended DART protein variants. Protein-RNA-bead complexes were incubated at room temperature for 30 min on a rotator, then moved to 4°C and incubated for 2 h with rotation.

Following the incubation period, RNA pulldown complexes were washed five times with 250 μl of Binding Buffer, each wash included a 3 min rotation at room temperature, to remove any unbounded purified DART protein, and supernatants were removed using a magnetic stand. Finally, 60 μl of Elution Buffer (50 mM Tris, pH 8, 200 mM NaCL, 2% (w/v) SDS, 1 mM Biotin) were added to each Protein-RNA-bead complex, mixed well by pipetting, and incubated at 60°C for 30 min. Eluents were collected following incubation using a magnetic stand. The eluents were then subjected to Western blotting for analysis.

### Synthesis of cDNA and Sanger Sequencing

Total RNA isolated from cells expressing DART protein was treated with DNase I (NEB) for 15 min at 37°C to remove possible DNA contamination, then RNA was isolated using ethanol precipitation. For *in vitro* DART-seq, total RNA was column purified, after incubation with purified DART protein. In both cases, cDNA was made using iScript Reverse Transcription Supermix (Bio-Rad). PCR amplification of the region surrounding selected mRNAs was carried out with CloneAmp HiFi PCR Mastermix (Takara). The resulting PCR product was gel-purified on a 1% agarose gel and gel extracted using the Qiaquick Gel Extraction Kit (Qiagen). Samples were submitted for Sanger sequencing (Genewiz) and % C2U was quantified using EditR software (Kluesner et al., 2018).

### Next-Generation Sequencing

All sequencing was performed by the Duke University Sequencing and Genomic Technologies Core facility. RNA samples purified from cellular DART-seq, and *in vitro* DART-seq as previously described were thawed on ice. For cellular DART-seq, 1 μg of RNA for each sample was used for sequencing library preparation using the NEBNext Ultra II Directional RNA Library Prep Kit for Illumina (NEB). For *in vitro* DART-seq, the entirety of RNA purified following incubation with purified DART protein was used for sequencing library preparation using the NEBNext Single Cell/Low Input RNA Library Prep Kit for Illumina (NEB). Before sequencing, all samples were barcoded using NEBNext Multiplex Oligos for Illumina (NEB), and their concentrations were quantified using Qubit Fluorometer (Thermo Fisher). Libraries were then sequenced on the NovaSeq 6,000.

### Identification of m^6^A Sites in Cellular DART-Seq

m^6^A sites were identified using the Bullseye analysis pipeline (Tegowski et al., 2022a). Bullseye is available on GitHub (https://github.com/mflamand/Bullseye). Raw sequencing data in fastq format were downloaded, and adapter sequences were trimmed using Flexbar (3.0.3). Sequences were aligned to the hg19 genome using NovoAlign. PCR duplicates were removed from the BAM files using Samtools (1.11). Then, using Bullseye, the parseBAM.pl script was used to parse the BAM files and create a counts matrix of the number of reads for each nucleotide at all positions with coverage. The Find_edit_site.pl script was then used to find C-to-U (or A-to-I) mutations with at least 10 reads of coverage, an edit ratio of 5–95%, and an edit ratio at least 1.2-fold higher than mutant control samples (APO1-YTH^mut^ or ADARcd-YTH^mut^), and at least 2 C-to-U (or A-to-I for cells expressing ADARcd-YTH^D422N^) editing events at a given site. Sites that were only found in one replicate of each DART protein variant were removed. For cells expressing DART protein with APO1 variants, those sites were further filtered to include only those occurring in an RAC (G/A-A-C) motif. Editing events observed when APOBEC1 alone was over-expressed in HEK293T cells (Meyer, 2019) were removed.

### Identification of m^6^A Sites With *in vitro* DART-Seq

m^6^A sites found by *in vitro* DART-seq were identified using Bullseye following a similar protocol as described above for cellular DART-seq. C-to-U mutations with at least 10 reads of coverage, an edit ratio of 5–95%, an edit ratio at least 1.2-fold higher than mutant control samples (APO1-YTH^mut^), and at least 2 C-to-U editing events at a given site were selected. Sites that were only found in one replicate of the APO1-YTH^D422N^ or APO1-YTH sample were removed. The remaining sites were further filtered to include only those occurring in an RAC motif. Editing events observed in any of the three replicates of samples treated with APOBEC1 alone were removed.

### Metagene Analysis

Metagene analysis was generated using metaPlotR (Olarerin-George and Jaffrey, 2017) with hg19 annotations as part of the computational pipeline in the Bullseye package.

### Relative Distance Analysis

Relative distance plots comparing the relative distance of either C-to-U editing events detected in cellular or *in vitro* DART-seq, or A-to-I editing events identified in cellular DART-seq with ADARcd-YTH^D422N^ against m^6^A sites called by miCLIP (Linder et al., 2015). Shuffle sites were generated using the Bullseye package. The program shuffle_sites.sh first finds all the exons of the transcripts containing edit sites. Then it shuffles the edit sites within these exons. The relative distance plots were generated in Rstudio using ggplot2 package.

### Cumulative Distribution Analysis

Cumulative distribution function plot and corresponding box plot were generated by comparing the C-to-U (A-to-I) editing percentage of DART-seq samples in Rstudion using the ggplot2 and the tidyverse package. A Wilcoxon Rank-Sum test was conducted in Rstudio using the tidyverse package to access statistical significance.

### Mass Spectrometry Analysis

Total RNA was isolated from either untreated or STM2457 treated (as described above) HEK293T cells using Trizol (Invitrogen) according to the manufacturer’s instructions and subsequently treated with DNase I (NEB) for 15 min at 37°C to remove possible DNA contamination. mRNA was then isolated with two rounds of purification using Dynabeads mRNA Purification Kit (Thermo Fisher). 200 ng of mRNA was digested with 2 U of Nuclease P1 (Sigma) in 50 ul nuclease free water with 2.5 mM ZnCl and 25 mM NaCl for 2 h at 37°C. Subsequently, mRNA samples were treated with 5 U of antarctic phosphatase (NEB) for 2 h at 37°C. Samples were then processed using the Xevo TQ-S mass spectrometry system.

### Comparison of Methylated Transcripts With REPIC Database

A text file containing the genomic coordinates, gene annotation, and dataset information for MeRIP peaks reported in HEK293T cells from 3 separate studies (Lichinchi et al., 2016; Meyer et al., 2012; Schwartz et al., 2014) was downloaded from the REPIC database. (https://repicmod.uchicago.edu/repic/download.php) (Liu et al., 2020). Gene names were then retrieved from the Ensembl Gene ID annotations. RNAs with called peaks in at least two of the three studies were then compared to the list of RNAs containing high-confidence m^6^A sites in the cellular DART-seq or *in vitro* DART-seq.

## Results

### Development of a DART Protein Variant With Improved m^6^A Recognition

Accurate detection of m^6^A sites by DART-seq relies on both m^6^A recognition and efficient deamination of m^6^A-adjacent cytidines. To achieve this, the DART fusion protein consists of the YTH domain of YTHDF2 tethered to the rat APOBEC1 cytidine deaminase (Meyer, 2019). However, it is possible that other variants of the YTH domain or alternative deaminase proteins may improve m^6^A detection. To explore this, we first tested other deaminase enzymes. This included members of the AID/APOBEC family of proteins known to act on RNA, as well as the rat APOBEC1 deaminase domain alone (Salter et al., 2016; Smith, 2017; Jin S. et al., 2020) (Supplementary Figure S1A). Each deaminase was fused to the YTH domain and expressed in HEK293T cells, followed by assessment of C-to-U deamination adjacent to m^6^A sites in a panel of mRNAs previously confirmed to contain m^6^A (Linder et al., 2015; Meyer, 2019) (Supplementary Figures S1B,C). Surprisingly, most of these proteins failed to show editing above background, and none of the proteins led to improved m^6^A detection compared to the original rat APO1-YTH fusion protein (Supplementary Figure S1C).

We next explored whether alternative YTH domains could improve DART-mediated detection of m^6^A. We tested three variants of the YTH domain: 1) the YTH domain of YTHDF1 (YTH^DF1^), which has a stronger affinity to some m^6^A-containing RNAs compared to the YTH domain of YTHDF2 (Zhu et al., 2014; Xu et al., 2015); 2) the YTH^DF1^ domain engineered to contain the D401N mutation, which lies in the m^6^A binding pocket and improves m^6^A recognition by 16-fold (Xu et al., 2015) (YTH^DF1(D401N)^); and 3) the YTH domain of YTHDF2 harboring an equivalent mutation, D422N (YTH^D422N^) (Supplementary Figure S1A).

**FIGURE 1:**
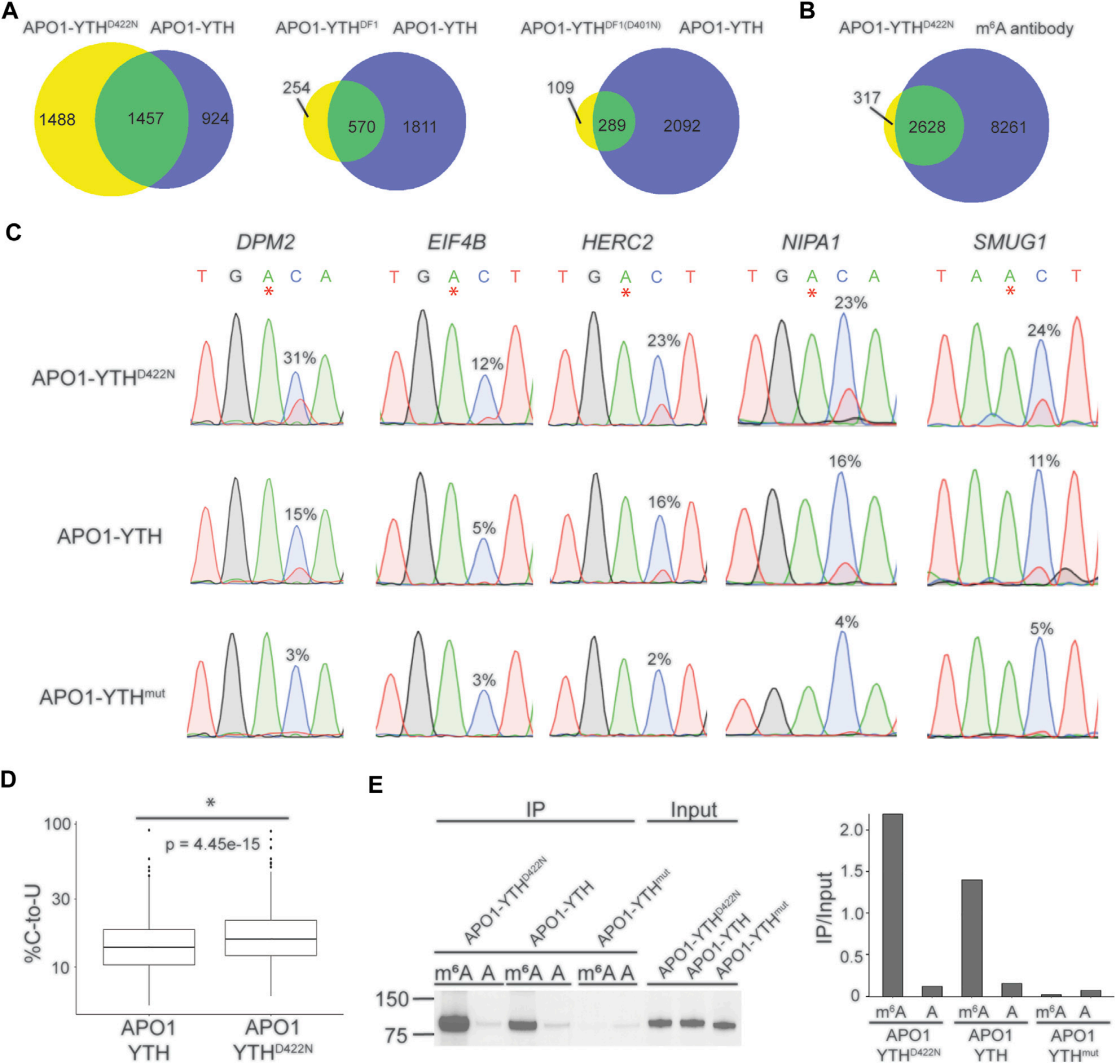
Identification of an improved variant of the DART fusion protein. **(A)** Comparison of methylated RNAs identified by cellular DART-seq using expression of either APO1-YTH^D422N^, APO1-YTH^DF1^, or APO1-YTH^DF1(D401N)^ in HEK293T cells. Venn diagrams compare each DART protein variant to the original APO1-YTH protein. **(B)** Overlap of methylated RNAs identified by cellular DART-seq using the APO1-YTH^D422N^ protein with those identified by antibody-based methods. **(C)** Sanger sequencing traces showing C-to-U editing adjacent to m^6^A sites in cells expressing APO1-YTH^D422N^, APO1-YTH, and APO1-YTH^mut^ for five mRNAs previously shown to contain m^6^A: *DPM2, EIF4B*, *HERC2*, *NIPA1*, and *SMUG1*. m^6^A sites are indicated by asterisks. C-to-U editing rate (%U) is indicated above the adjacent cytidine. Data are representative of three biological replicates. **(D)** Box plot showing the global C-to-U editing percentage of all sites common to HEK293T cells expressing APO1-YTH^D422N^ or APO1-YTH. **(E)** Western blot following RNA pulldown assays using purified DART proteins and bait RNAs. APO1-YTH^D422N^ exhibits improved binding to m^6^A compared to APO1-YTH.

Each YTH variant was fused to rat APO1 and overexpressed in HEK293T cells. We then performed DART-seq to assess the ability of each DART protein variant to detect m^6^A sites. C-to-U editing events in cells expressing each DART protein were enriched within the vicinity of the stop codon, consistent with the distribution of m^6^A (Supplementary Figure S1D). We then identified m^6^A sites from each dataset using Bullseye, a pipeline that we previously developed for analysis of DART-seq data (Tegowski et al., 2022a). Comparison of all DART protein variants showed that APO1-YTH^D422N^ identified the greatest number of methylated RNAs, which overlapped well with methylated RNAs identified by antibody-based approaches (Figures 1A,B, Supplementary Table S1). The sites that were identified by APO1-YTH^D422N^ but not by APO1-YTH exhibited a distribution in transcripts that matches that of m^6^A and were found in RNAs identified by antibody-based methods, suggesting that these were not caused by false-positives (Supplementary Figures S1E,F). Additionally, C-to-U editing rates (% C2U) of m^6^A sites identified by APO1-YTH^D422N^ were higher than those of the original DART protein in a panel of selected mRNAs, suggesting increased sensitivity of APO1-YTH^D422N^ for detecting m^6^A (Figures 1C,D). Consistent with this, RNA pulldown assays revealed that APOBEC1-YTH^D422N^ has improved binding to methylated RNA compared to the wild type YTHDF2 YTH domain (Figure 1E). Thus, the YTH^D422N^ domain enables improved m^6^A recognition and better sensitivity for m^6^A detection using DART-seq.

### ADARcd-Mediated DART-Seq Is an Alternative Method for Identifying Methylated RNAs

Using a cytidine deaminase as the catalytic protein in DART-seq enables nucleotide-resolution m^6^A mapping since nearly all m^6^A sites are followed by a cytidine (Wei and Moss, 1977; Dominissini et al., 2012; Meyer et al., 2012; Meyer, 2019). However, the adenosine deaminase ADAR offers an alternative approach for the identification of methylated RNAs through targeted A-to-I editing. This is analogous to the TRIBE method in which the ADAR catalytic domain (ADARcd) is fused to an RNA-binding protein of interest and RNA targets are identified by A-to-I editing (Mcmahon et al., 2016). The HyperTRIBE method, which utilizes ADARcd containing a hyperactivating E488Q mutation, further provides increased sensitivity (Rahman et al., 2018; Xu et al., 2018). We therefore wondered whether using the hyperactive ADARcd in place of APO1 would enable DART-seq to identify methylated RNAs with greater sensitivity.

**FIGURE 2:**
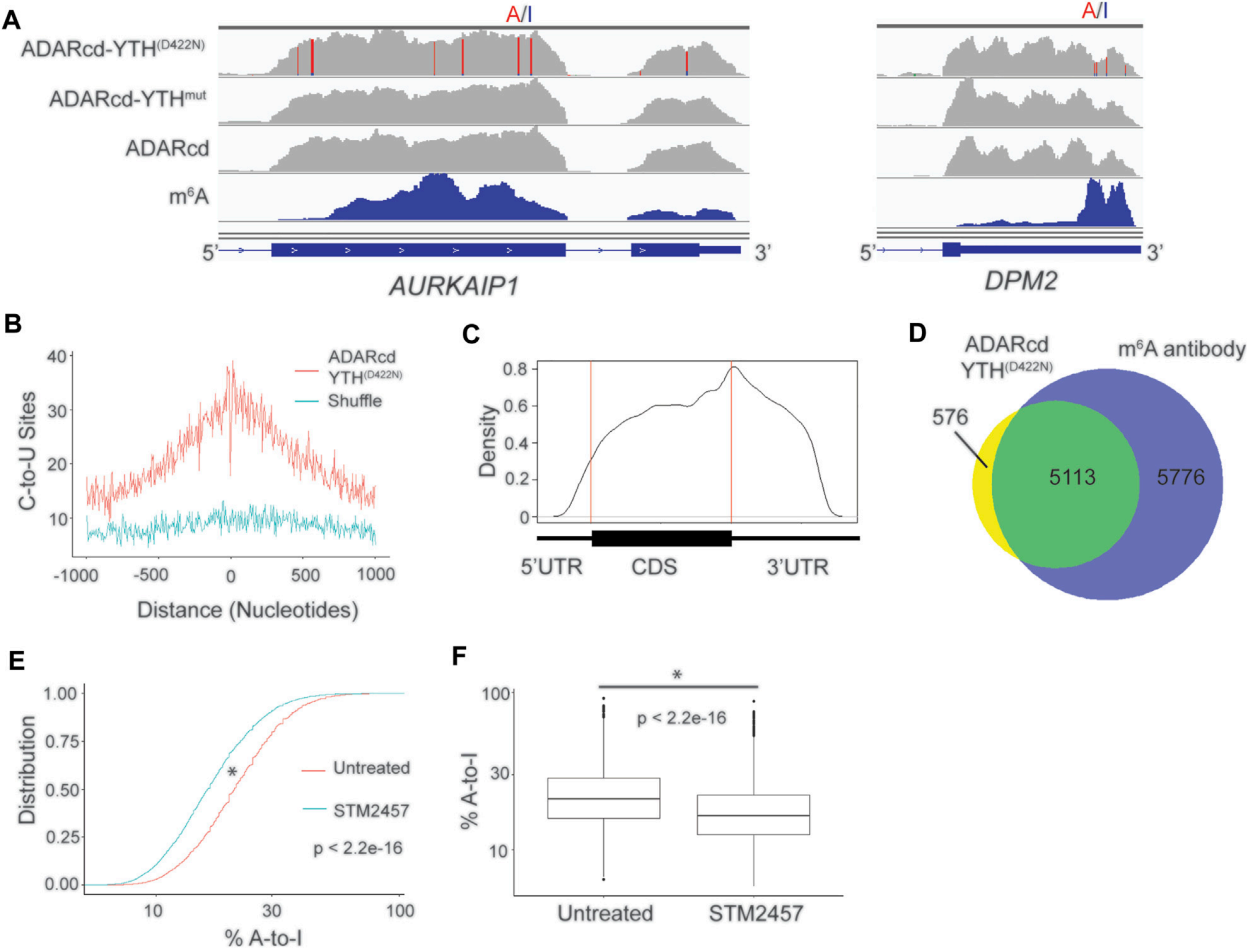
ADARcd can be used as an alternative to APO1 to identify methylated RNAs with DART-seq. **(A)** Genome browser tracks showing two methylated mRNAs, *AURKAIP1* and *DPM2*, in HEK293T cells expressing ADARcd-YTH^D422N^, ADARcd-YTH^mut^, or ADARcd alone. A-to-I editing found in at least 10% of the reads are indicated by red/blue coloring. m^6^A peaks identified by MeRIP (Meyer et al., 2012) is indicated in the bottom blue track. **(B)** Absolute distance plot showing the distance between A-to-I edit sites identified by ADARcd-YTH^D422N^ and m^6^A sites identified by miCLIP (Linder et al., 2015). **(C)** Metagene plot showing the distribution of A-to-I edit sites found in cells expressing ADARcd-YTH^D422N^. **(D)** Venn diagram showing overlap between methylated RNAs identified by cellular DART-seq from HEK293T cells expressing ADARcd-YTH^D422N^ and methylated RNAs identified by antibody-based profiling (Meyer et al., 2012; Schwartz et al., 2014; Lichinchi et al., 2016). **(E)** Cumulative distribution plot (left) of %A-to-I for sites identified by ADARcd-YTH^D422N^ in untreated and STM2457 treated HEK293T cells. **(F)** Box plot showing the global A-to-I editing percentage of all sites common to both untreated and STEM2457 treated HEK293T cells expressing ADARcd-YTH^D422N^. A Wilcoxon Rank-Sum test was conducted to access statistical significance.

To test this, we fused the hyperactive ADARcd to the YTH^D422N^ domain (ADARcd-YTH^D422N^) and expressed it in HEK293T cells for 24 h followed by RNA-seq. In parallel, we expressed ADARcd alone and ADARcd-YTH^mut^ as controls. We then modified the Bullseye pipeline to identify A-to-I editing events which were absent in cells expressing ADARcd alone and which were enriched in ADARcd-YTH^D422N^-expressing cells compared to ADARcd-YTH^mut^-expressing cells (Figure 2A, Supplementary Figure S2A; Methods). Overall, we observed consistent A-to-I editing of RNAs across biological replicates, indicating the reproducibility of RNA targeting by ADAR-YTH^D422N^ (Supplementary Figure S2B). We identified a total of 21,717 A-to-I editing sites in 5,689 RNAs that were common to two out of three biological replicates and used these sites for downstream analyses (Supplementary Table S3). These sites were enriched in the vicinity of m^6^A and had a distribution in mRNAs which matches that of m^6^A, indicating that YTH^D422N^ can effectively target ADARcd to m^6^A (Figures 2B,C). Additionally, there was a high degree of overlap between methylated RNAs identified by ADARcd-YTH^D422N^ and antibody-based methods (Figure 2D). To confirm that A-to-I editing events observed with ADARcd-YTH^D422N^ were m^6^A dependent, we expressed ADARcd-YTH^D422N^ in HEK293T cells treated with the METTL3 inhibitor STM2457 (Yankova et al., 2021) (Supplementary Figure S2C). We then performed RNA-seq and examined the effect of STM2457 treatment on A-to-I editing transcriptome-wide. We found that STM2457 treatment led to a global reduction in the total number of A-to-I editing events (21,718 and 16,250 A-to-I sites for untreated and STM2457 treated samples, respectively), among common sites identified between treated and untreated samples, 75% of the same sites identified showed reduced %A-to-I editing.(Figures 2E,F, Supplementary Table S3). Altogether, these data confirm that A-to-I editing induced by ADAR-YTH^D422N^ is METTL3-dependent and indicate that ADAR can be used in place of APO1 to identify m^6^A-containing RNAs by DART-seq.

We next compared the ability of ADARcd-YTH^D422N^ and APO1-YTH^D422N^ to identify methylated RNAs. Although there was high overlap of methylated RNAs identified by both methods, there were many more transcripts identified by ADAR-YTH^D422N^ (Supplementary Figure S3A). Consistent with this, there were also more A-to-I editing sites than C-to-U editing sites identified transcriptome-wide (21,718 and 6,042, for ADARcd-YTH^D422N^ and APO1-YTH^D422N^, respectively) (Supplementary Table S1). The methylated RNAs uniquely identified by ADAR-YTHD^422N^ showed good agreement with those identified by antibody-based methods, and A-to-I editing sites in transcripts had a distribution that matches m^6^A, suggesting that these sites were not caused by non-specific editing (Supplementary Figures S3B,C).

Since the majority of m^6^A sites are found within the GAC consensus sequence, most A-to-I editing caused by ADAR-YTH^D422N^ does not occur adjacent to m^6^A, and the Bullseye pipeline therefore does not filter sites based on the RAC consensus. In contrast, C-to-U editing caused by APO1-YTH^D422N^ can occur adjacent to m^6^A, and Bullseye filters sites to include only those that occur in the RAC consensus. Removing this filter leads to a much greater number of C-to-U sites (12,129 sites compared to 6,042 sites), but it is still fewer than the number of A-to-I sites of ADAR-YTH^D422N^ (Supplementary Table S3). In addition, comparing the methylated RNAs identified by these non-RAC-filtered sites with those identified by ADAR-YTH^D422N^ shows a greater number of methylated RNAs identified by ADAR-YTH^D422N^ (5,689 compared to 4,083, respectively), suggesting that it has greater sensitivity for m^6^A detection (Supplementary Figure S3D). Thus, both APO1-YTH^D422N^ and ADAR-YTH^D422N^ are effective methods for identifying methylated RNAs in cells, with ADAR-YTH^D422N^ offering slightly increased sensitivity and APO1-YTH^D422N^ having the distinct advantage of identifying m^6^A sites with single-nucleotide resolution.

### *In vitro* DART-Seq Detects m^6^A Transcriptome-Wide From Low Amounts of Input RNA

One limitation of DART-seq is that it requires expression of the DART fusion protein in cells or tissues of interest. This may not be desirable or even possible in some cell types, such as those from difficult-to-target tissues or human samples. To overcome this limitation, we previously demonstrated that *in vitro* DART-seq is capable of profiling m^6^A transcriptome-wide (Meyer, 2019). However, this strategy used a crude preparation of the DART fusion protein and failed to identify m^6^A sites with the same efficiency as cellular DART-seq.

**FIGURE 3:**
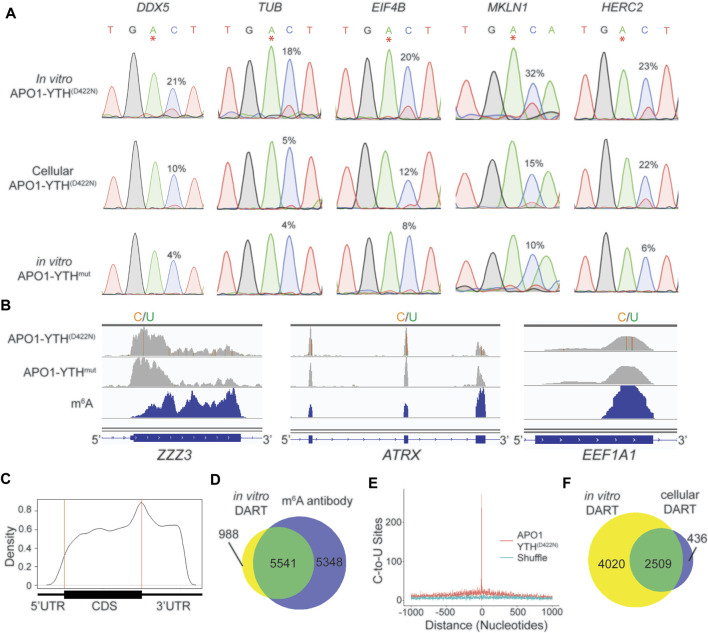
*In vitro* DART-seq identifies m^6^A transcriptome-wide. **(A)** Comparison of C-to-U editing rates in methylated mRNAs obtained by *in vitro* DART-seq and cellular DART-seq. Sanger sequencing traces show C-to-U editing adjacent to m^6^A sites in a panel of five methylated mRNAs: *DDX5, TUB*, *EIF4B*, *MKLN1*, and *HERC2*. m^6^A sites are indicated by asterisks. C-to-U editing rate (%U) is indicated above the adjacent cytidine. Data Representative of three biological replicates. **(B)** Genome browser tracks of *in vitro* DART-seq data showing C-to-U editing in three representative mRNAs: *ZZZ3, ATRX, and EEF1A1*. C-to-U editing found in at least 10% of the reads is indicated by green/yellow coloring. m^6^A peaks identified by MeRIP (Meyer et al., 2012) is indicated in the bottom blue track. **(C)** Metagene analysis of m^6^A sites identified by *in vitro* DART-seq using the APO1-YTH^D422N^ protein. **(D)** Venn diagram showing the overlap between methylated RNAs identified by *in vitro* DART-seq filtered against the APO1-YTH^mut^ negative control and methylated RNAs identified by antibody-based methods (Meyer et al., 2012; Schwartz et al., 2014; Lichinchi et al., 2016). **(E)** Absolute distance plot showing the distance of C-to-U editing sites identified by *in vitro* DART-seq relative to m^6^A sites identified by miCLIP (Linder et al., 2015). m^6^A sites are centered at position 0. **(F)** Venn diagram showing the overlap between methylated RNAs identified by *in vitro* DART-seq compared to methylated RNAs found by cellular DART-seq.

We therefore sought to develop an improved version of *in vitro* DART-seq which can be used to profile m^6^A in any sample of interest while maintaining the high sensitivity and low input requirements of cellular DART-seq. We first generated purified APO1-YTH^D422N^ and APO1-YTH^mut^ proteins using a bacterial expression system (Supplementary Figure S4A) (Tegowski et al., 2022b). We then performed *in vitro* DART assays with HEK293T cell RNA followed by RT-PCR and Sanger sequencing to assess editing adjacent to m^6^A sites in a panel of methylated mRNAs. APO1-YTH^D422N^ produced robust C-to-U editing adjacent to m^6^A sites, an effect that was greatly reduced when APO1-YTH^mut^ was used (Figure 3A). Optimization of *in vitro* DART conditions showed that higher concentrations of APO1-YTH^D422N^ protein led to higher C-to-U editing rates but decreased enrichment in % C2U relative to APO1-YTH^mut^ samples, indicating that oversaturation with too much protein can likely increase the rate of false-positives (Supplementary Figure S4B). Similarly, longer incubation times led to higher editing rates but lower % C2U enrichment for APO1-YTH^D422N^ relative to APO1-YTH^mut^ (Supplementary Figure S4C). Thus, higher protein: RNA ratios and extended assay times may improve the detection of low-abundance m^6^A sites, but careful calibration relative to the APO1-YTH^mut^ control condition is needed to avoid false-positives.

We next assessed the ability of *in vitro* DART-seq to identify m^6^A sites transcriptome-wide. We performed *in vitro* DART-seq using 50 ng of total HEK293T cell RNA from three biological replicates. In parallel, we performed *in vitro* DART-seq using APO1-YTH^mut^ and then used Bullseye to identify m^6^A sites that were enriched in APO1-YTH^D422N^ samples relative to APO1-YTH^mut^ samples (Figure 3B, Supplementary Table S2). There was high overlap of methylated RNAs identified among biological replicates, indicating the reproducibility of the *in vitro* DART-seq approach (Supplementary Figure S4D). Additionally, C-to-U editing sites showed a distribution within transcripts that matches m^6^A, and methylated RNAs identified by *in vitro* DART-seq showed a high degree of overlap with those identified by antibody-based methods (Figures 3C,D). C-to-U editing sites from *in vitro* DART-seq were also found at C-to-T mutations in miCLIP data, indicating that *in vitro* DART-seq can successfully identify m^6^A sites transcriptome-wide (Figure 3E).

We next compared the methylated RNAs identified by APO1-YTH^D422N^ with *in vitro* DART-seq to those of cellular DART-seq. The majority (85.2%) of methylated RNAs identified by cellular expression of APO1-YTH^D422N^ were also identified *in vitro*. However, *in vitro* DART-seq identified a much greater number of methylated RNAs (Figure 3F, Supplementary Table S2). C-to-U editing sites uniquely identified by *in vitro* DART-seq had a distribution that matches m^6^A and occurred at C-to-T mutations sites previously called by miCLIP (Supplementary Figures S4E,F). This suggests that the greater number of methylated RNAs identified by *in vitro* DART-seq relative to cellular DART-seq is not caused by false positives and instead likely reflects greater sensitivity of the *in vitro* DART-seq approach.

Finally, to determine whether the increased sensitivity of APO1-YTH^D422N^ compared to APO1-YTH that we observed in cells was also recapitulated *in vitro*, we performed *in vitro* DART-seq using APO1-YTH. We found that APO1-YTH^D422N^ identified more m^6^A sites and methylated RNAs than APO1-YTH (Supplementary Figure S5A, Supplementary Table S2), and C-to-U editing sites identified by APO1-YTH^D422N^ also had significantly higher % C2U values than sites identified by APO1-YTH (Supplementary Figures S5B,C). Similar to cellular DART-seq, the sites uniquely identified by APO1-YTH^D422N^ using *in vitro* DART-seq had a distribution that matches m^6^A and were enriched at C-to-T mutations from miCLIP data (Linder et al., 2015), indicating that they are not due to false-positives (Supplementary Figures S5D,E). Altogether, we demonstrate *in vitro* DART-seq as a highly sensitive antibody-independent m^6^A detection method.

### YTH Domain Blocking Improves *in vitro* DART-Seq m^6^A Detection Specificity

The use of APO1-YTH^mut^ to control for non-specific deamination helps ensure the identification of high-confidence m^6^A sites. However, because the YTH^mut^ domain retains low-level m^6^A binding (Figure 1D), it is possible that some m^6^A sites are eliminated from DART-seq datasets when filtering against APO1-YTH^mut^ as a control. We therefore sought to develop alternative methods for eliminating false-positive site calls while minimizing false-negatives.

**FIGURE 4:**
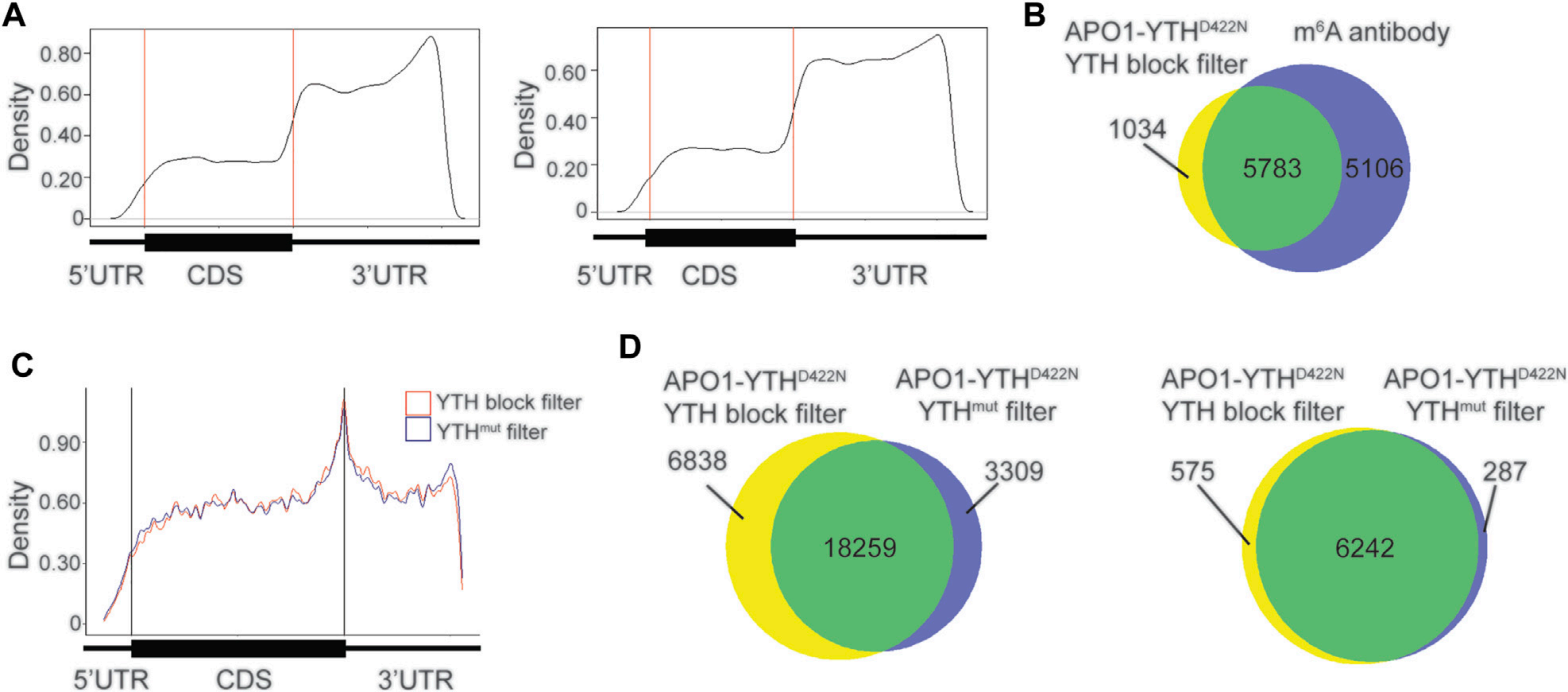
Blocking with the YTH domain minimizes false positives with *in vitro* DART-seq. **(A)** Metagene analysis of C-to-U editing sites in mRNAs identified with *in vitro* DART-seq using the APO1-YTH^D422N^ protein after pre-incubation with the YTH domain (left) or the APO1-YTH^mut^ protein (right). **(B)** Venn diagram showing the overlap between methylated RNAs identified by *in vitro* DART-seq with APO1-YTH^D422N^ filtered by YTH blocking and methylated RNAs identified by antibody-based methods (Meyer et al., 2012; Schwartz et al., 2014; Lichinchi et al., 2016). **(C)** Metagene analysis showing the distribution of C-to-U editing sites in mRNAs after filtering of *in vitro* DART-seq data against by YTH blocking (blue) or by APO1-YTH^mut^ (red). **(D)** Venn diagram of C-to-U edit sites induced by *in vitro* DART-seq with APO1-YTH^D422N^, filtered by use of either YTH blocking or APO1-YTH^mut^ (left). Venn diagram of methylated RNA identified by *in vitro* DART-seq with APO1-YTH^D422N^, filtered by use of either YTH blocking or APO1-YTH^mut^ (right).

We tested whether blocking the DART protein from binding to m^6^A sites could be an effective alternative to the use of APO1-YTH^mut^. To do this, we purified the YTH domain (see Methods) and subjected HEK293T cell RNA to *in vitro* DART-seq using a modified protocol in which the RNA sample was pre-incubated with the YTH domain before addition of APO1-YTH^D422N^ (see Methods). m^6^A sites were called by establishing a minimum editing enrichment threshold in the APO1-YTH^D422N^ condition relative to the YTH blocking condition, similar to what was done when using the APO1-YTH^mut^ control. C-to-U editing events that remained after YTH blocking showed a distribution distinct from that of m^6^A and were enriched in the distal 3′UTR (Figure 4A). This was similar to the distribution of sites identified in the APO1-YTH^mut^ condition, suggesting that both methods can be used to identify false-negative sites.

We next assessed whether the YTH blocking strategy improves the m^6^A detection accuracy of *in vitro* DART-seq compared to the APO1-YTH^mut^ control method. To do this, we filtered C-to-U editing sites in the APO1-YTH^D422N^ dataset by their % C2U enrichment relative to either the YTH blocking dataset or the APO1-YTH^mut^ dataset (see Methods). There was a high degree of overlap in the m^6^A sites and methylated RNAs identified by both datasets and a similar distribution of m^6^A sites within RNAs (Figures 4B,C). Interestingly, using APO1-YTH^mut^ as a negative control led to the exclusion of more sites (21,568 total sites identified when filtering against APO1-YTH^mut^ vs. 25,097 total sites identified when filtering against the YTH domain blocking dataset) (Figure 4D, Supplementary Table S4). Comparison of the sites unique to each filtering method showed a similar enrichment which matched that of m^6^A, and the RNAs containing these sites were also identified by miCLIP, suggesting that these unique sites are not false-positives (Supplementary Figures S6A,B). Interestingly, the % C2U values of sites that were unique to the YTH blocking method of filtering were significantly lower than those of the APO1-YTH^mut^ method of filtering (Supplementary Figures S6C,D). This suggests that identifying sites by filtering against the YTH blocking dataset enables the detection of lower abundance m^6^A sites compared to the APO1-YTH^mut^ method. This is consistent with the low-level m^6^A binding of APO1-YTH^mut^, which likely leads to editing adjacent to some m^6^A sites and therefore their exclusion when using this method as a negative control. In addition, the unique sites identified with YTH blocking filtering showed an increased number of C-to-U edit sites adjacent to previously identified m^6^A sites by miCLIP (Supplementary Figure S6E). Altogether, these data suggest that both APO1-YTH^mut^ and YTH domain blocking can serve as effective controls against which to filter *in vitro* DART-seq data for elimination of false-positives. Both strategies perform similarly well, but YTH domain blocking enables slightly more sites to be identified, which likely reflect low-abundance m^6^A sites.

## Discussion

In this study, we present an improved version of DART-seq which utilizes a variant of the APO1-YTH fusion protein containing an engineered D422N mutation within the YTH domain. This variant exhibits improved m^6^A recognition compared to the original APO1-YTH fusion protein and enables detection of m^6^A transcriptome-wide with slightly greater sensitivity. Surprisingly, our attempts to optimize the editing domain of the DART fusion protein by using alternative cytidine deaminase proteins failed to identify a variant capable of editing RNAs adjacent to m^6^A sites. This may reflect the requirement for a specific structural conformation of the fusion protein to permit access of the editing domain to m^6^A-adjacent cytidines. Future studies examining the structure of APO1-YTH in complex with RNA would undoubtedly shed more light on how the fusion protein interacts with methylated RNA substrates to target cytidine residues that occur adjacent to m^6^A.

Although the cytidine deaminase variants that we tested failed to improve DART protein editing, we discovered that swapping ADARcd for APO1 led to efficient deamination of adenosines in methylated RNAs. The resulting A-to-I editing sites are enriched near m^6^A, although because m^6^A occurs within a RAC consensus sequence, these sites are not immediately adjacent to m^6^A. Thus, unlike APO1-YTH^D422N^, ADARcd-YTH^D422N^ cannot identify m^6^A sites with single-nucleotide resolution. However, direct comparison of both fusion proteins in cells indicated that ADARcd-YTH^D422N^ identifies a greater number of methylated RNAs, indicating that it has increased sensitivity for identifying methylated RNAs at the whole transcript level. However, one consideration when using this approach is that ADARcd is known to exhibit preferential editing of double-stranded RNA regions (Eggington et al., 2011; Jin H. et al., 2020); thus, ADARcd-YTH^D422N^ may miss some methylated RNAs that lack suitable double-stranded regions in near m^6^A sites. Such issues will be important to consider for each individual study when deciding which DART fusion protein to use. In theory, it should also be possible to co-express both APO1-YTH^D422N^ and ADARcd-YTH^D422N^ at the same time in cells and identify transcripts with both A-to-I and C-to-U editing. Such a strategy would minimize the false-positives of both approaches and still provide single-nucleotide resolution m^6^A mapping.

In addition to improving the cellular DART-seq method, we also developed an *in vitro* DART-seq approach which enables m^6^A mapping from any sample of interest without the need for overexpression of the DART fusion protein. We demonstrate that *in vitro* DART-seq performs comparably to cellular DART-seq and that it can be used to accurately profile m^6^A sites from low amounts of input material. Since a major limitation of m^6^A mapping studies has been the requirement for large amounts of purified RNA, we anticipate that *in vitro* DART-seq will now enable m^6^A mapping studies that were not previously possible, such as those that utilize human tissues or frozen samples.

An important component of both cellular and *in vitro* DART-seq is the use of controls to help eliminate false-positive sites. This includes identifying sites that are edited by the APO1 or ADARcd proteins alone and eliminating them from consideration. We have also traditionally used APO1-YTH^mut^ as a negative control. Although the YTH^mut^ domain exhibits reduced m^6^A recognition, it still retains some m^6^A binding ability and therefore contributes to low-level C-to-U deamination when fused to APO1 (Meyer, 2019; Tegowski et al., 2022a). The Bullseye pipeline therefore uses thresholds based on % C2U enrichment relative to APO1-YTH^mut^ to identify high-confidence m^6^A sites. However, this may lead to the elimination of some true m^6^A sites which retain sufficient levels of editing by APO1-YTH^mut^. We have mitigated this to some extent here by developing a YTH pre-blocking method for *in vitro* DART-seq, which eliminates the need for the APO1-YTH^mut^ control. We find that the YTH blocking approach enables the identification of slightly more m^6^A sites which may otherwise be filtered out using the APO1-YTH^mut^ strategy as a control. Thus, for *in vitro* DART-seq, employing a pre-blocking step with the YTH domain alone may be preferred. Other methods for eliminating false-positives, such as the recently developed use of modification-free libraries (Zhang et al., 2021), are alternative strategies which may further increase the accuracy of the *in vitro* DART-seq method.

In summary, we have developed an improved version of the DART fusion protein and a suite of new methods related to the DART-seq approach which will facilitate more accurate and sensitive m^6^A detection. The development of *in vitro* DART-seq in particular provides a method for transcriptome-wide m^6^A mapping in nearly any sample of interest and overcomes the need for large amounts of input material that are required for most m^6^A mapping approaches. Therefore, we anticipate that this method will enable future studies of m^6^A in tissues or cell types that were otherwise not amenable to m^6^A profiling.

## Data Availability

The datasets presented in this study can be found in online repositories. The names of the repository/repositories and accession number(s) can be found below: NCBI’s Gene Expression Omnibus (GEO) under accession code GSE196416.

## Supplementary Material

Supplementary Figure S1 Comparison of different DART protein variants. **(A)** Schematic showing the domain composition of all DART protein variants tested. **(B)** Western blot showing the expression of DART protein variants after expression in HEK293T cells. Results are representative of three biological replicates for each DART protein variant. **(C)** Sanger sequencing traces showing C-to-U editing adjacent to m6A sites found by cellular DART-seq with DART variants in panel **(A)** for five selected mRNAs: DPM2, EIF4B, HERC2, NIPA1, and SMUG1. Most variants failed to show editing above background (>5%). rZDD-YTH demonstrated C-to-U editing, but it was lower than that of APO1-YTHD422N. Results are representative of three biological replicates. **(D)** Metagene analysis of C-to-U editing sites identified by DART-seq in HEK293T cells expressing the indicated DART protein variant. **(E)** Metagene analysis of C-to-U editing events unique to APO1-YTHD422N that were not identified by cellular DART-seq with APO1-YTH. **(F)** Venn diagram showing the overlap between methylated RNAs identified by C-to-U editing events unique to APO1-YTHD422N that are not found in APO1-YTH, and methylated RNAs found by antibody-based profiling (Lichinchi et al., 2016, Meyer et al., 2012, Schwartz et al., 2014).

Supplementary Figure S2 Validation of m6A-methylated RNAs identified by cellular DART-seq with ADARcd-YTHD422N. **(A)** Genome browser track showing the JUN mRNA in HEK293T cells expressing ADARcd-YTHD422N. A-to-I editing found in at least 10% of the reads are indicated by red/blue coloring (left). Expanded region of JUN (right) shows A-to-I editing (marked by arrow in both panels) in proximity to an m6A site previously identified by miCLIP (Linder et al., 2015). **(B)** Venn diagram of m6A-containing RNAs identified by each of the three biological replicates of cells expressing ADARcd-YTHD422N. **(C)** Mass spectrometry data examining the ratio between m6A/A in mRNA purified from untreated HEK293T cells or HEK293T cells treated with STM2457. The graph shows the average of two biological replicates in each condition and the error bar represents the standard deviation.

Supplementary Figure S3 Validation of m6A-dependent editing by ADARcd-YTHD422N. **(A)** Venn diagram of methylated RNAs identified by cellular DART-seq with ADARcd-YTHD422N compared to those identified by APO1-YTHD422N. **(B)** Venn diagram of methylated RNAs identified uniquely by cellular DART-seq with ADARcd-YTHD422N and not identified by cellular DART-seq with APO1-YTHD422N, compared to methylated RNAs found by miCLIP (Linder et al., 2015). **(C)** Metagene analysis of A-to-I editing sites in methylated transcripts uniquely identified in ADARcd-YTHD422N DART-seq data and not in APO1-YTHD422N DART-seq data. **(D)** Venn diagram of methylated RNAs identified by cellular DART-seq with ADARcd-YTHD422N compared to those identified by APO1-YTHD422N without the RAC filter.

Supplementary Figure S4 Optimization and validation of *in vitro* DART-seq as a global m6A profiling method. **(A)** Coomassie stain showing the purification of the APO1-YTHD422N (left) and APO1-YTH (right) proteins. **(B)** Quantification of Sanger sequencing data showing C-to-U editing rate ratio (APO1-YTHD422N/APO1-YTHmut) for cytidine residues adjacent to m6A sites in three representative mRNAs: ACTB, DPM2, and HERC2. *In vitro* DART assays were performed using x nanograms of total HEK293T cell RNA and 250 nanograms of APO1-YTHD422N incubated at 37°C for the indicated time points. *n*=2 biological replicates; error bars = standard deviation. **(C)** Quantification of Sanger sequencing data as in **(B)** for *in vitro* DART assays using the indicated concentration of APO1-YTHD422N and APO1-YTHmut protein incubated with 50 nanograms of total HEK293T cell RNA at 37°C for 4 h. *n*=2 biological replicates; error bars = standard deviation. **(D)** Overlap of methylated RNAs identified in each of the three biological replicates of *in vitro* DART-seq using APO1-YTHD422N. **(E)** Metagene plot of C-to-U editing sites in methylated transcripts uniquely found by *in vitro* DART-seq with APO1-YTHD422N compared to cellular DART-seq with the same protein variant. **(F)** Absolute distance plot showing the distance of C-to-U edit sites in methylated RNAs identified by APO1-YTHD422N that were not identified by cellular DART-seq with the same protein variant, and m6A sites identified by miCLIP (Linder et al., 2015).

Supplementary Figure S5 Comparison of APO1-YTHD422N and APO1-YTH with *in vitro* DART-seq. **(A)** Comparison of methylated RNAs identified by *in vitro* DART-seq using APO1-YTHD422N or APO1-YTH. **(B)** Cumulative distribution of C-to-U editing rates for sites in *in vitro* DART-seq data using the APO1-YTHD422N or APO1-YTH protein. **(C)** Box plot showing the editing percentage of C-to-U sites identified by *in vitro* DART-seq using APO1-YTHD422N or APO1-YTH both filtered by APO1-YTHmut. **(D)** Metagene analysis of C-to-U editing sites uniquely identified by APO1-YTHD422N but not identified by APO1-YTH. **(E)** Absolute distance plot showing the distance of C-to-U editing sites uniquely identified by APO1-YTHD422N compared to APO1-YTH from m6A sites identified by miCLIP (Linder et al., 2015). m^6^A sites are centered at position 0.

Supplementary Figure S6 Comparison of YTH blocking and APO1-YTHmut as controls for *in vitro* DART-seq. **(A)** Distribution of C-to-U editing sites in methylated RNAs identified by APO1-YTHD422N filtered by the YTH blocking dataset but not when filtered by APO1-YTHmut (left); vice-versa (right). **(B)** Venn diagram showing overlap of methylated RNAs identified by APO1-YTHD422N after filtering by YTH blocking (left) or APO1-YTHmut (right) with methylated RNAs identified by miCLIP (Linder et al., 2015). **(C)** Cumulative distribution of C-to-U editing rates of sites identified by *in vitro* DART-seq using APO1-YTHD422N and filtered by YTH blocking APO1-YTHmut. **(D)** Box plot showing C-to-U editing rates of sites identified by *in vitro* DART-seq using purified APO1-YTHD422N filtered by the YTH blocking dataset or by APO1-YTHmut dataset. **(E)** Absolute distance plot showing the distance of C-to-U editing sites identified by APO1-YTHD422N filtered by YTH blocking and not identified by APO1-YTHmut relative to m6A sites identified by miCLIP (Linder et al., 2015). m6A sites are centered at position 0.

Supplementary Table S1 Sites identified by DART protein variants using cellular DART-seq in HEK293T cells. Listed are the genome coordinates of DART-seq C-to-U editing sites identified from cells expressing DART protein variants APO1-YTHD422N, APO1-YTH, APO1-YTH(DF1), and APO1-YTH(DF1-D401N). Also indicated is the proportion of C-to-U editing (U/C).

Supplementary Table S2 Sites identified by APO1-YTH and APO1-YTHD422N with *in vitro* DART-seq. Listed are the genome coordinates of DART-seq C-to-U editing sites identified from *in vitro* DART-seq assay using purified APO1-YTH protein or purified APO1-YTHD422N protein. Also indicated are the proportion of C-to-U editing (U/C).

Supplementary Table S3 A-to-I edit sites identified by cellular DART-seq with ADARcd-YTHD422N. Listed are the genome coordinates of m6A dependent A-to-I edit sites identified from HEK293T cells expressing ADARcd-YTHD422N and from HEK293T cells treated with STM2547 expressing ADARcd-YTHD422N. Also indicated are the proportion of A-to-I editing and the region of distribution of these sites.

Supplementary Table S4 C-to-U editing events induced by APO1-YTHD422N *in vitro* DART-seq filtered by YTH blocking technique.
