# Supplementary figures and images for: Improved Methods for Deamination-Based m6A Detection
Source: Front Cell Dev Biol. 2022 Apr 27;10:888279. doi: 10.3389/fcell.2022.888279 (PMC9092492; doi:10.3389/fcell.2022.888279)

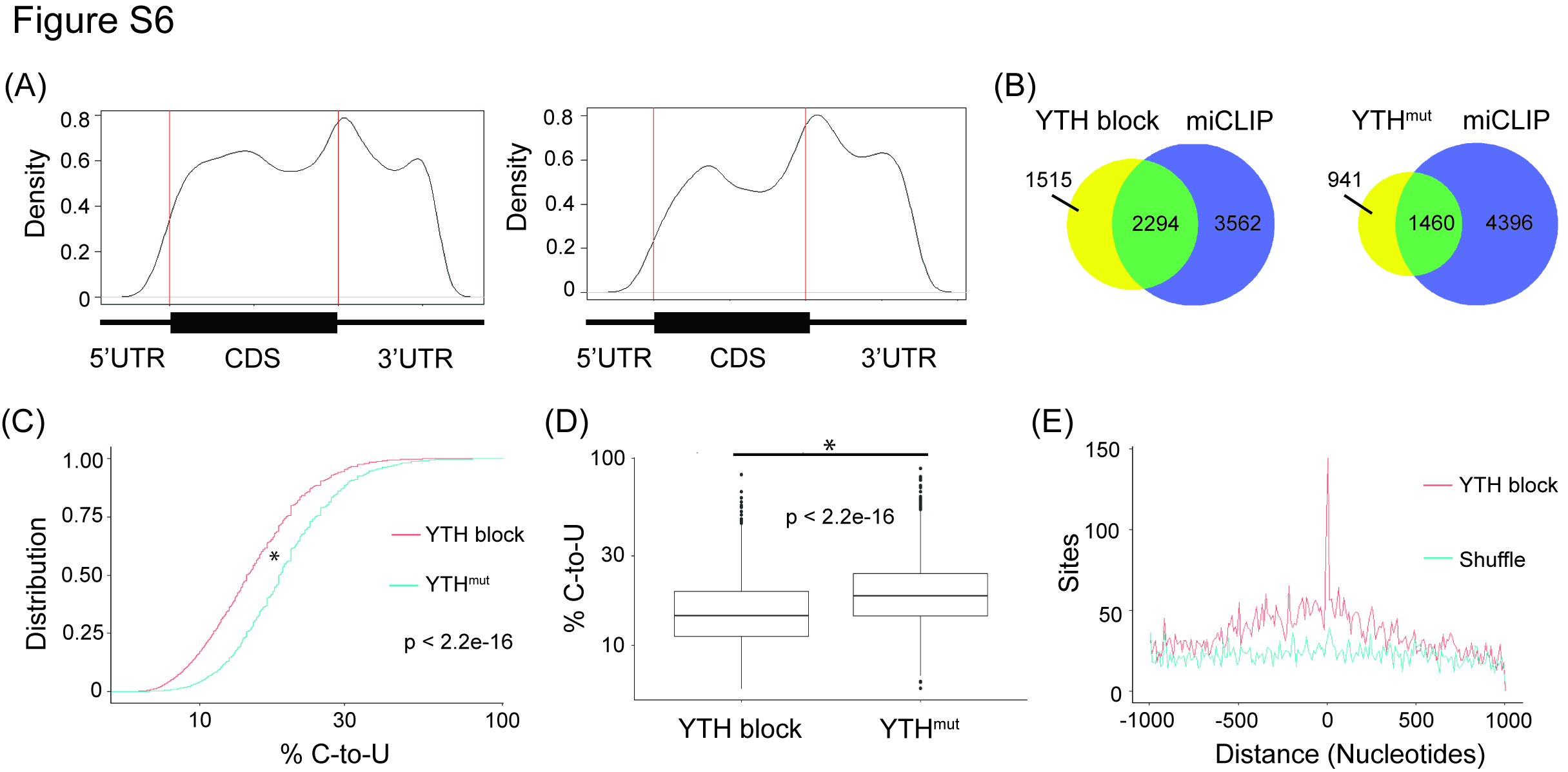

Supplement: Supplementary file 3 [file Image6.TIF]

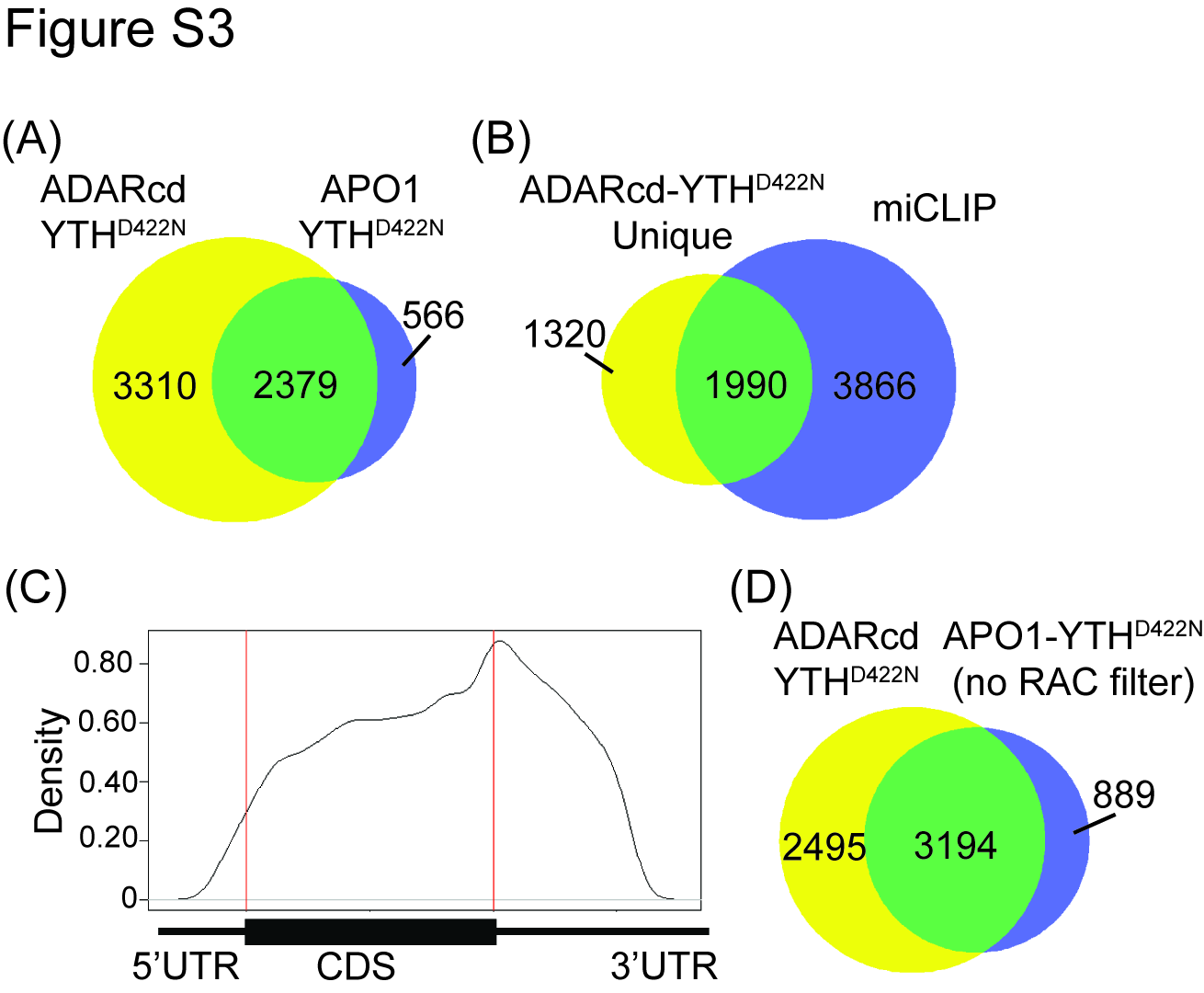

Supplement: Supplementary file 4 [file Image3.TIF]

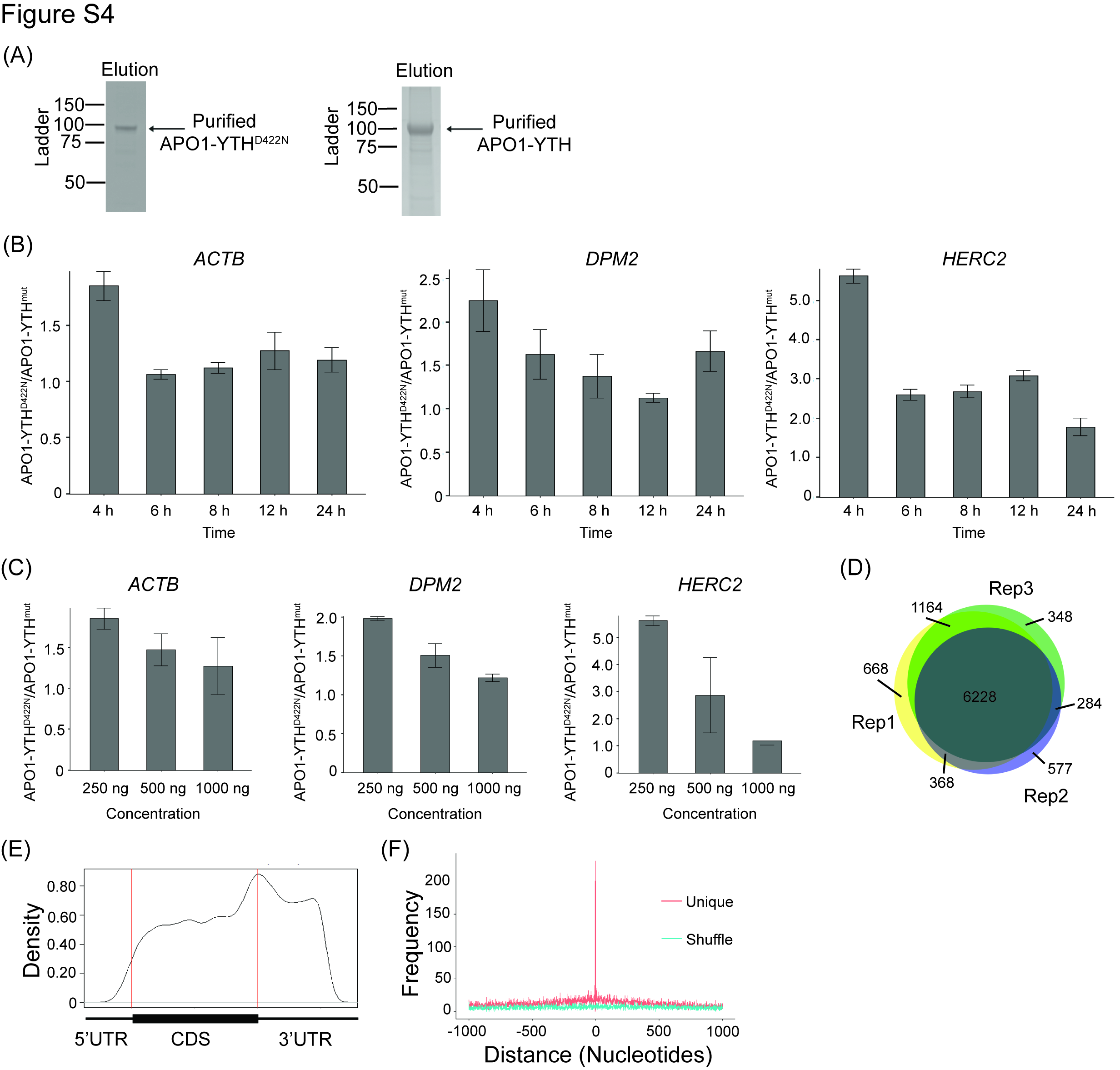

Supplement: Supplementary file 5 [file Image4.TIF]

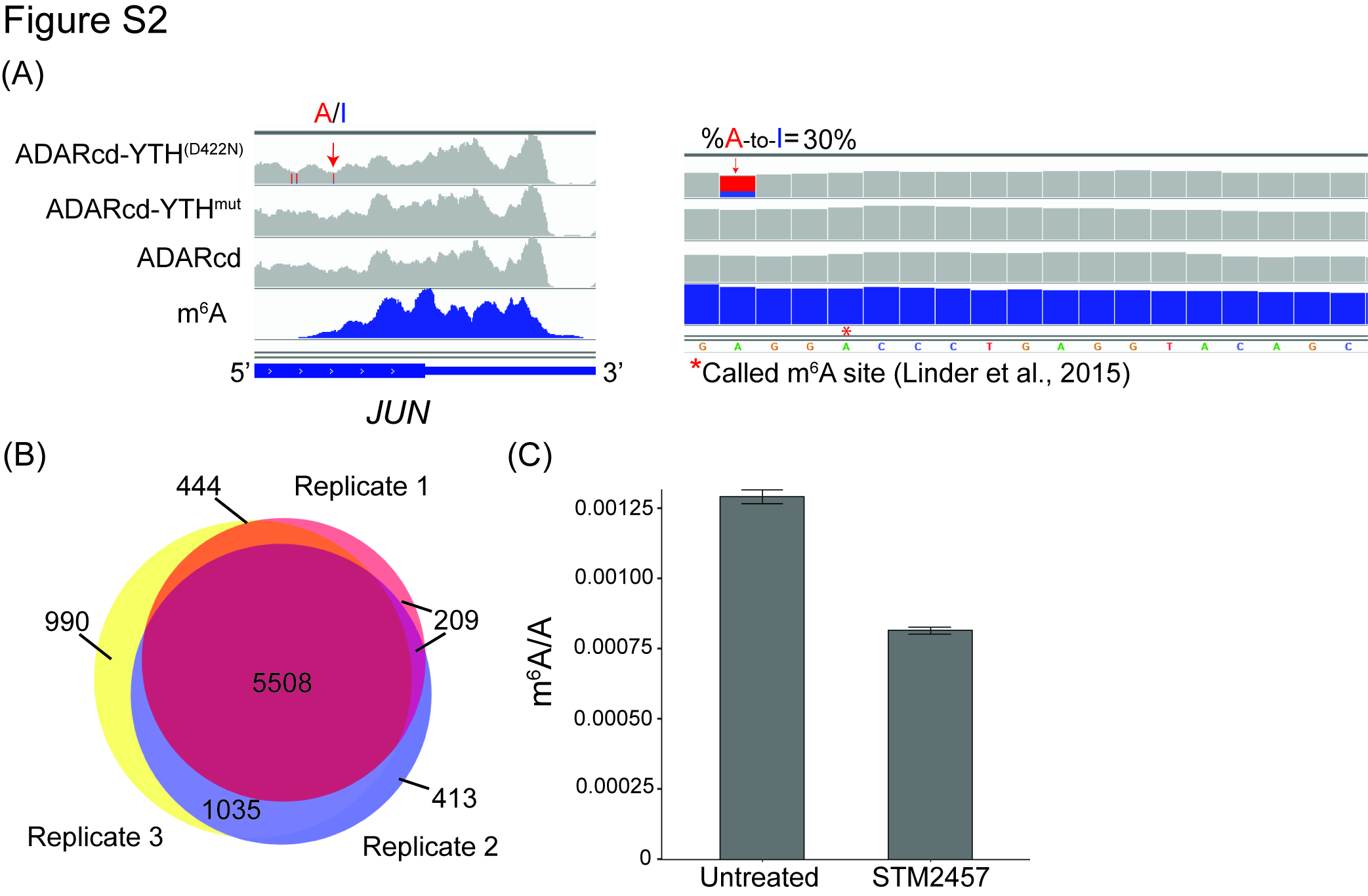

Supplement: Supplementary file 6 [file Image2.TIF]

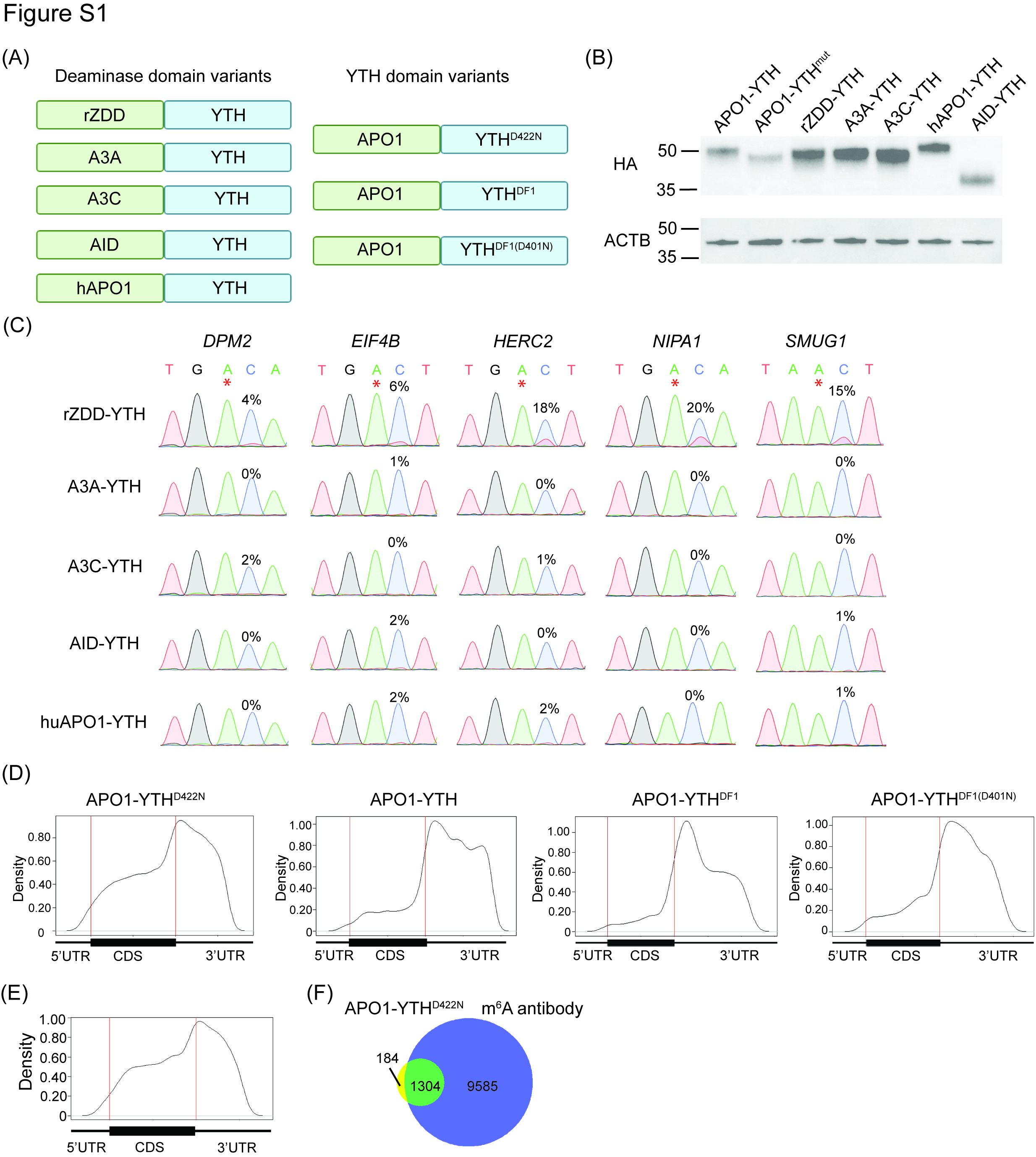

Supplement: Supplementary file 7 [file Image1.TIF]

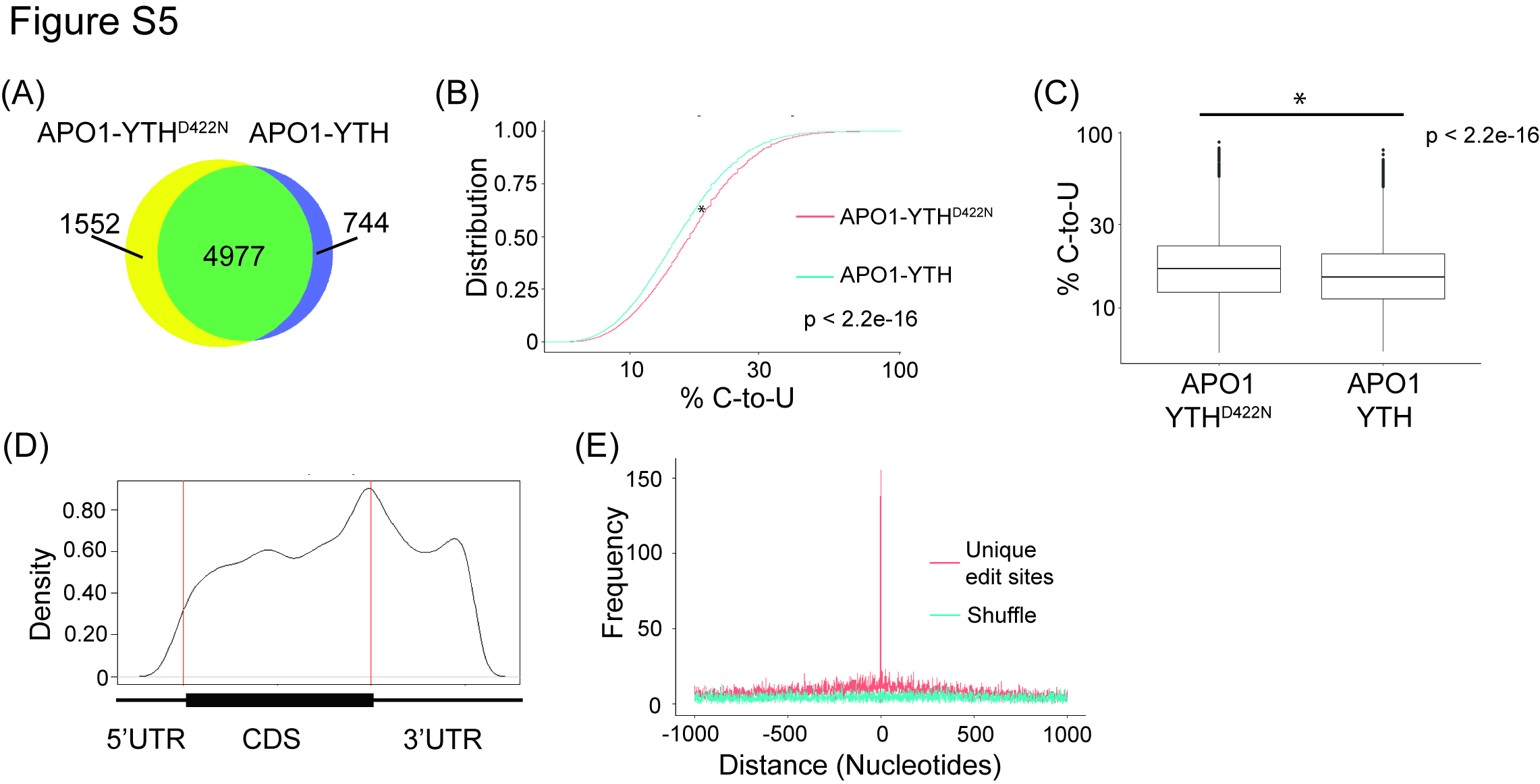

Supplement: Supplementary file 10 [file Image5.TIF]
